# The Histomorphologic Profile of Skin Diseases in Kuwait

**DOI:** 10.7759/cureus.48729

**Published:** 2023-11-13

**Authors:** Rawan Almutairi, Humoud Al-Sabah

**Affiliations:** 1 Dermatopathology, As'ad Al-Hamad Dermatology Center, Kuwait City, KWT

**Keywords:** psoriasis, lichen planus, mycosis fungoides, biopsy, kuwait, dermatology

## Abstract

Background

Although dermatological disorders are common in all countries, their spectrum varies greatly, with a wide histological variation. This study aimed to investigate the frequency and spectrum of different histopathological patterns of skin lesions in relation to age and gender in Kuwait.

Methodology

This was a retrospective descriptive study. Skin biopsy samples collected over a five-year period from 2018 to 2022 at the dermatopathology department of a tertiary dermatology center in Kuwait were included in this study. The distribution of lesions according to age and gender was analyzed.

Results

Of the 1,796 skin tissues reviewed, the ages ranged from one month to 93 years, with a mean age of 38.9 years. A female predominance was noted, with a female-to-male ratio of 1.8:1. Most patients belonged to the 30-39-year age group. The most frequent diagnostic categories were neoplasms and papulosquamous diseases. The five most common diseases were psoriasis, lichen planus, mycosis fungoides, benign melanocytic nevus, and epidermal inclusion cysts. The most commonly encountered diseases were similarly distributed according to gender (p > 0.05).

Conclusions

Neoplasms and papulosquamous lesions dominated this investigation. Therefore, understanding the genetic and environmental factors that contribute to psoriasis is crucial for developing effective treatment strategies and comprehensively managing the condition. Additionally, the community should be educated to prevent repeated unprotected ultraviolet light sun exposure and early diagnosis of any suspicious lesions to reduce the prevalence of neoplastic skin diseases. Histopathological research on cutaneous lesions is rare, with none reported from Kuwait. Our histopathology-based retrospective analysis provides a baseline for population-specific skin disease studies.

## Introduction

Skin disorders are common and range from simple acne to potentially fatal melanomas. While many cutaneous problems are intrinsic to the skin, others are manifestations of underlying medical conditions. Consequently, the skin is considered a window for identifying a wide range of diseases [1]. Skin biopsy is an essential diagnostic procedure that assists dermatologists in confirming the diagnosis, staging lesions, and discovering many etiological factors of dermatological diseases [2]. Despite the wide range of histological findings associated with skin illnesses, the clinical presentation is limited to only a few alterations, such as hyper or hypopigmentation, macules, papules, and nodules, necessitating histopathological confirmation [3]. This study aims to investigate the frequency and spectrum of different histopathological patterns of skin lesions in relation to age and gender at a tertiary dermatology center in Kuwait.

## Materials and methods

This was a retrospective review of all skin biopsies received and reported by the Department of Dermatopathology at a tertiary dermatology center in Kuwait between 2018 and 2022. All pathological reports with final diagnoses of skin biopsies that were diagnosed by a board-certified dermatopathologist were retrieved. Demographic data such as age and gender were also retrieved from the pathology reports of these patients. Final diagnoses of the skin diseases were then categorized, similar to other histopathological studies, into the following categories: benign and malignant neoplasms, papulosquamous, connective tissue diseases, dermatitis and eczema, pigmentary disorders, vesiculobullous, infections, hair disorders, metabolic, panniculitis, and psychodermatological diseases. Other non-specific skin changes were grouped under the category of others and miscellaneous. Data were analyzed using the frequency and percentage. Data analysis was performed using SPSS version 22 (IBM Corp., Armonk, NY, USA). The inclusion criterion was samples retrieved from the dermatopathology department that clearly demonstrated any specific pathology during the study period. Similarly, skin biopsies that did not show definite signs of any specific pathology, or inadequate samples, were excluded. The Kuwaiti Ministry of Health’s Standing Committee for the Coordination of Health and Medical Research granted ethical approval number for this study (approval number: 2255-2023).

## Results

A total of 1,796 cases were recorded in the dermatopathology department between 2018 and 2022. Of the 1,796 cases, 1,146 (63.8%) were female, and 650 (36.2%) were male. The female-to-male ratio was 1.8:1. The patients’ ages ranged from 0.1 and 93 years. In general, the mean age of all cases was 38.9 ± 19.3. The mean ages of the female and male patients were 39.1 and 38.3, respectively. The highest number of patients (n = 377; 21%) was seen in the 30-39-year age group. The distribution of all cases according to age and gender is presented in Table 1. The most frequent diagnostic categories were neoplasms and papulosquamous. Table 2 shows the frequencies and percentages of different categories of skin diseases.

**Table 1:** The distribution of cases according to age and gender. The data has been represented as (n) number of patients and %.

Age groups ( years)	Male		Female		Total	
	n	%	n	%	n	%
0.1–9	44	2.4	45	2.5	89	5.0
10–19	85	4.7	125	7.0	210	11.7
20–29	122	6.8	184	10.2	306	17.0
30–39	140	7.8	237	13.2	377	21.0
40–49	122	6.8	208	11.6	330	18.4
50–59	89	5.0	116	6.5	205	11.4
60–69	40	2.2	105	5.8	145	8.1
70–79	34	1.9	60	3.3	94	5.2
80–89	19	1.1	16	0.9	35	1.9
90–100	3	0.2	2	0.1	5	0.3
Total	698	38.9	1098	61.1	1796	100.0

**Table 2:** Frequencies and percentages of different categories of skin diseases. The data has been represented as (n) number of patients and %.

Diagnosis	n = 1,796	(%)
Neoplasms	542	30.2
A. Benign	371	20.7
Epidermal tumors	147	8.2
Dermal tumors	93	5.2
Appendageal tumors	52	2.9
Soft tissue tumors	79	4.4
B. Malignant	171	9.5
Papulosquamous lesions	288	16.0
Connective tissue diseases	138	7.7
Dermatitis and eczema	129	7.2
Disorders of pigmentation	125	7.0
Vesicobullous disorders	101	5.6
Infections	106	5.9
A. Bacterial	47	2.6
B. Fungal	21	1.2
C. Viral infections	17	0.9
D. Parasitic	14	0.8
Hair disorders	47	2.6
Urticaria and related diseases	34	1.9
Metabolic disorders	14	0.8
Neutrophilic dermatosis	6	0.3
Panniculitis	6	0.3
Psychodermatological disease	3	0.2
Others/Miscellaneous disorders	257	14.3

The five most commonly encountered diseases were psoriasis (n = 116/1,796, 6.46%), lichen planus (n = 99/1,796, 5.51%), mycosis fungoides (n = 94/1,796, 5.23%), benign melanocytic nevus (n = 67/1,796, 3.7%), and epidermal inclusion cysts (n = 63/1,796, 3.51%). These diseases accounted for 24.4% (n = 439/1,796) of the observed cases. These diseases are major problems between the ages of 20 and 50 years. The most commonly encountered diseases were similarly distributed according to gender (p > 0.05).

## Discussion

The youngest patient evaluated in this study was one month old and the oldest was 93 years old. Other studies conducted in India and Nepal reported patients’ ages up to the seventh and eighth decades, respectively [4,5]. Skin lesions were more prevalent in females (n = 1,146/1,796, 63.8%). Similar observations were made by other studies, with values of 55.7% and 61.3%, respectively [6,7]. However, male predominance was observed in another study (68%) [8]. The greater number of skin diseases in women may be attributable to their heightened awareness of skin issues for cosmetic reasons as well as their heightened sensitivity to health-related issues in general. In addition, certain skin diseases exhibit sex-specific differences that contribute to female predominance, particularly connective tissue diseases, such as cutaneous lupus erythematosus.

The neoplasm category represented the highest number of cases (n = 542/1,796, 30.18%), which included benign and malignant cases. This high percentage demonstrates the crucial role of biopsies in distinguishing benign from malignant lesions among suspicious lesions. Epidermal inclusion cysts and pilar cysts are among the most frequently encountered benign tumors. Our findings are comparable to those in Saudi Arabia [9], Turkey [6], and Iran [10].

Mycosis fungoides, a variety of cutaneous T-cell lymphomas, was our study’s most commonly diagnosed malignant lesion type, representing almost half of the malignant diseases (n = 94/171, 55%). Men and women were afflicted in approximately equal numbers (46 and 48 cases, respectively). Our findings differed from those of Saudi Arabia, where mycosis fungoides was the third most frequent malignancy after basal cell carcinoma and squamous cell carcinoma [11]. In Saudi Arabia, males are twice as likely as females to develop mycosis fungoides, typically occurring between the ages of 30 and 40 [12]. Environmental exposures, such as the westernization of lifestyle, and prolonged exposure to pesticides may be responsible for the increase in incidence [13]; however, more research is required to determine the precise causes of the rising incidence of the disease.

Basal cell carcinoma was the second most common malignant lesion observed in our study (n = 48/171, 28.1%). A study conducted in Nepal also showed that basal cell carcinoma was the second most common malignant tumor (30%) after squamous cell carcinoma (42.5%) [14], while in Saudi Arabia it was the most common malignant tumor [11]. It appears on the epidermis layer of the head and neck skin, which is the most sun-exposed area of the body. Therefore, it makes sense that ultraviolet radiation from the sun could be a potential cause of basal cell carcinoma in this region. The community can be educated to minimize repeated, unprotected exposure to sunlight after 10 AM and to practice sun safety measures, such as the use of sunblocks.

Papulosqamous diseases represented 16% of the study cases (n = 288/1,796), with the domination of psoriasis and lichen planus at 6.5% (n = 116/1,796) and 5.5% (n = 99/1,796), respectively. Our results are in line with those from Turkey, where psoriasis and lichen planus were the most prevalent papulosquamous diseases, representing 9.2% of their cases [6]. The preponderance of our psoriasis biopsy cases was erythrodermic psoriasis. The two most prevalent causes of erythrodermic psoriasis were withdrawal of oral corticosteroids and withdrawal of excessive topical corticosteroids. Several factors play a role in the development and exacerbation of psoriasis. Factors such as stress, smoking, alcohol consumption, and obesity have been associated with an increased risk of developing psoriasis. In particular, obesity has been identified as a common risk factor for psoriasis and can modify the effects of genetic factors on psoriasis development [15].

In this study, eczema and dermatitis accounted for 7.18% (n = 129/1,796) of all the cases. In a previous study from another region of Kuwait, eczema and dermatitis accounted for 23.7% of all cases [16], contrary to our observations. This is because the diagnosis of eczema and dermatitis is based primarily on the patient’s medical history and physical examination. In doubtful cases, histopathological examination is typically encouraged.

Our study showed a high predominance of melanocytic nevus among pigmented disorders (n = 67/125, 53.6% and n = 67/1796, 3.73%) of all cases studied, similar to Saudi Arabia, where melanocytic nevus was the most common pigmentary lesion (11.8%) [9]. These primarily represented benign melanocytic nevi. Given that malignant melanoma, although rare, is one of the differential diagnoses for melanocytic nevus, the high number of cases demonstrates the importance of performing a skin biopsy in suspicious pigmented lesions to rule out malignant melanoma. Vitiligo was the least frequently reported pigmentary disorder (n = 3/1,796, 0.17%) in our study. This could be attributed to the fact that vitiligo is primarily diagnosed based on the patient’s medical history and physical examination, and a biopsy is rarely administered for diagnostic purposes. In case of doubt, a histopathological examination is recommended.

Vesicobullous lesions were observed in 5.62% of all skin lesions (n = 101/1,796). Bullous pemphigoid was most common (n = 39/101, 38.6%), followed by pemphigus vulgaris (n = 25/101, 24.8%). These findings were comparable to the findings of other studies that documented that 2.7% and 4.8% of all dermatoses were vesiculobullous lesions [17,18]. Around 60% of our patients with bullous pemphigoid are in and above the seventh decade of life, without a gender predilection. However, there are rare case reports of bullous pemphigoid detected in children and adolescents [19]. The number of cases of bullous pemphigoid is on the rise, particularly among the elderly. The specific causes of this increase are not fully understood; however, age-related immune system changes and environmental factors are possible explanations.

Among bacterial diseases, leprosy (n = 17/47, 36.2%) and syphilis (n = 16/47, 34.0%) were the two most common infections. Lepromatous leprosy is the most common form of leprosy detected in our study. Most of our patients with leprosy were from southern Asia, and the prevalence of leprosy remains high in this part of the world [20]. Studies conducted in Southern Asia showed a high prevalence of leprosy in the infectious disease category, and our results were comparable with those of other studies [17,21].

## Conclusions

Several skin lesions had varied age distributions. Geographic distribution, genetics, socioeconomic status, culture, and personal cleanliness affect the prevalence of skin diseases. Neoplasms and papulosquamous lesions dominated this investigation. Therefore, understanding the genetic and environmental factors that contribute to psoriasis is crucial for developing effective treatment strategies and comprehensively managing the condition. Additionally, the community should be educated to prevent repeated unprotected ultraviolet light sun exposure and early diagnosis of any suspicious lesions to reduce the prevalence of neoplastic skin diseases. Histopathological research on cutaneous lesions is rare, and none has been discovered in Kuwait. Our histopathology-based retrospective analysis provides a baseline for population-specific skin disease studies. We recommend additional national and worldwide histopathological studies.

## References

[1] Li AW, Yin ES, Stahl M, Kim TK, Panse G, Zeidan AM, Leventhal JS (2017). The skin as a window to the blood: cutaneous manifestations of myeloid malignancies. Blood Rev.

[2] Rajaratnam R, Smith AG, Biswas A, Stephens M (2009). The value of skin biopsy in inflammatory dermatoses. Am J Dermatopathol.

[3] Rapini RP (2021). Practical Dermatopathology.

[4] Adhikari RC, Shah M, Jha AK (2019). Histopathological spectrum of skin diseases in a tertiary skin health and referral centre. J Pathol Nepal.

[5] Bharadwaj V, Sudhakar R, Srikanth Reddy K (2020). Histopathological spectrum of dermatological lesions- a retrospective study. J Evid Based Med Healthc.

[6] Bilgili ME, Yildiz H, Sarici G (2013). Prevalence of skin diseases in a dermatology outpatient clinic in Turkey. A cross-sectional, retrospective study. J Dermatol Case Rep.

[7] El-Khateeb EA, Imam AA, Sallam MA (2011). Pattern of skin diseases in Cairo, Egypt. Int J Dermatol.

[8] Grover S, Ranyal RK, Bedi MK (2008). A cross section of skin diseases in rural Allahabad. Indian J Dermatol.

[9] Albasri AM, Ansari IA (2019). The histopathological pattern of benign and non-neoplastic skin diseases at King Fahad Hospital, Madinah, Saudi Arabia. Saudi Med J.

[10] Baghestani S, Zare S, Mahboobi AA (2005). Skin disease patterns in Hormozgan, Iran. Int J Dermatol.

[11] Albasri AM, Borhan WM (2018). Histopathological pattern of skin cancer in Western region of Saudi Arabia. An 11 years experience. Saudi Med J.

[12] AlGhamdi KM, Arafah MM, Al-Mubarak LA, Khachemoune A, Al-Saif FM (2012). Profile of mycosis fungoides in 43 Saudi patients. Ann Saudi Med.

[13] Morales-Suárez-Varela MM, Olsen J, Johansen P (2005). Occupational exposures and mycosis fungoides. A European multicentre case-control study (Europe). Cancer Causes Control.

[14] Thapa R, Gurung P, Hirachand S, Shrestha SB (2018). Histomorphologic profile of skin tumors. JNMA J Nepal Med Assoc.

[15] Zeng J, Luo S, Huang Y, Lu Q (2017). Critical role of environmental factors in the pathogenesis of psoriasis. J Dermatol.

[16] Al-Haqan E, El-Gamal A, Al-Owaidi H (2012). Pattern of dermatological disorders in Al-Adan Hospital: southern region of Kuwait. Gulf J Dermatol Venereol.

[17] Narang S, Jain R (2015). An evaluation of histopathological findings of skin biopsies in various skin disorders. Ann Pathol Lab Med.

[18] George V (2020). A histopathological study of skin biopsy specimens in a tertiary care hospital with a keynote on clinicopathological correlation. Ann Pathol Lab Med.

[19] Taquin H, Chiaverini C, Lacour JP (2016). Spectrum of clinical responses to therapies in infantile bullous pemphigoid. Pediatr Dermatol.

[20] Scollard DM, Adams LB, Gillis TP, Krahenbuhl JL, Truman RW, Williams DL (2006). The continuing challenges of leprosy. Clin Microbiol Rev.

[21] Gupta P, Karuna V, Grover K (2018). Verma N, The histopathological spectrum of skin diseases with emphasis on clinicopathological correlation: a prospective study. J Diagn Pathol Oncol.

